# A Review of Piezoelectric and Magnetostrictive Biosensor Materials for Detection of COVID‐19 and Other Viruses

**DOI:** 10.1002/adma.202005448

**Published:** 2020-11-24

**Authors:** Fumio Narita, Zhenjin Wang, Hiroki Kurita, Zhen Li, Yu Shi, Yu Jia, Constantinos Soutis

**Affiliations:** ^1^ Department of Frontier Sciences for Advanced Environment Graduate School of Environmental Studies Tohoku University Aoba‐yama 6‐6‐02 Sendai 980‐8579 Japan; ^2^ Department of Materials Processing Graduate School of Engineering Tohoku University Aoba‐yama 6‐6‐02 Sendai 980‐8579 Japan; ^3^ College of Automation Engineering Nanjing University of Aeronautics and Astronautics 29 Jiangjun Avenue Nanjing 211106 China; ^4^ Department of Mechanical Engineering University of Chester Thornton Science Park, Pool Lane Chester CH2 4NU UK; ^5^ School of Engineering and Applied Science Aston University Birmingham B4 7ET UK; ^6^ Aerospace Research Institute The University of Manchester Oxford Road Manchester M13 9PL UK

**Keywords:** artificial intelligence, biosensors, data analytics, detection properties, electromagneto‐mechanical design, Internet of Things, machine learning, piezoelectric/magnetostrictive materials, virus

## Abstract

The spread of the severe acute respiratory syndrome coronavirus has changed the lives of people around the world with a huge impact on economies and societies. The development of wearable sensors that can continuously monitor the environment for viruses may become an important research area. Here, the state of the art of research on biosensor materials for virus detection is reviewed. A general description of the principles for virus detection is included, along with a critique of the experimental work dedicated to various virus sensors, and a summary of their detection limitations. The piezoelectric sensors used for the detection of human papilloma, vaccinia, dengue, Ebola, influenza A, human immunodeficiency, and hepatitis B viruses are examined in the first section; then the second part deals with magnetostrictive sensors for the detection of bacterial spores, proteins, and classical swine fever. In addition, progress related to early detection of COVID‐19 (coronavirus disease 2019) is discussed in the final section, where remaining challenges in the field are also identified. It is believed that this review will guide material researchers in their future work of developing smart biosensors, which can further improve detection sensitivity in monitoring currently known and future virus threats.

## Graphical abstract

Piezoelectric and magnetostrictive biosensor materials are presented and discussed. It is found that these advanced materials show great potential for application in the detection of various viruses. Progress related to COVID‐19 (coronavirus disease 2019) and the way to new and emerging sensors for virus detection for home application or wearability are considered.

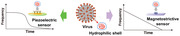

## 1 Introduction

**Figure 1:**
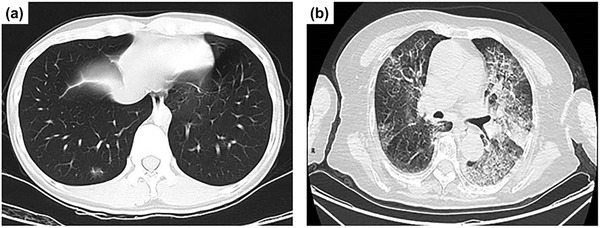
Chest computerized tomography images of patients with novel coronavirus pneumonia. a) Patchy ground‐glass‐like density change from a 33 year old patient who developed a mild illness after exposure at work and b) bilateral diffuse thickening of interlobular septa with network‐like densities, bronchiolar thickening, and consolidation of the left lower lobe in an 83 year old patient. Reproduced with permission.^[^ ^1^ ^]^ Copyright 2020, Wiley Periodicals, Ltd.

In December 2019, an acute febrile illness with a severe respiratory distress syndrome began to appear, and an evolving situation was reported involving infection with a novel coronavirus, named severe acute respiratory syndrome coronavirus 2 (SARS‐CoV‐2).^[^ ^1^ ^]^ Most patients with SARS‐CoV‐2 experience fever with or without chills, chest tightness, dry cough, and shortness of breath, while developing patchy to diffuse infiltration of the lungs, as shown radiographically in **Figure** **1**. Identifying and monitoring SARS‐CoV‐2 infection has become crucially important. A recent projection of the transmission dynamics of SARS‐CoV‐2^[^ ^2^ ^]^ showed that longitudinal serological studies are desperately needed to determine the extent and duration of immunity to SARS‐CoV‐2. Even in the event of apparent elimination, maintenance of SARS‐CoV‐2 surveillance is still needed because of a possible resurgence of contagion.

**Figure 2:**
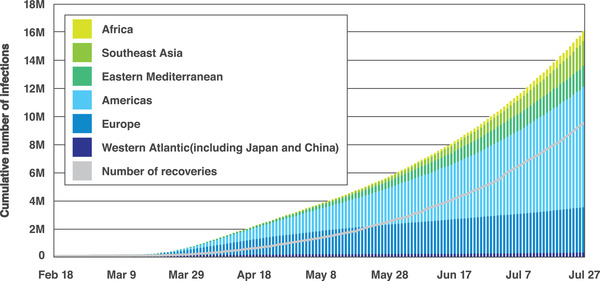
Accumulated confirmed SARS‐CoV‐2 infections as of July 27, 2020. Reproduced with permission.^[^ ^4^ ^]^ Copyright 2020, Johns Hopkins University.

In the past, notable viruses have emerged suddenly from obscurity or anonymity, provoking concern from the point of view of immunology regarding their sustained epidemic transmission in naive human populations. More than 70% of these infections have been zoonotic, entering either directly from wild animal reservoirs or indirectly via an intermediate domestic animal host.^[^ ^3^ ^]^ Ebola virus, avian influenza, human immunodeficiency virus (HIV), and SARS are all examples of zoonoses that have emerged from wild animals, presenting an increasingly serious threat to human health and economies worldwide. **Figure** **2** shows the trend in the number of people infected with SARS‐CoV‐2.^[^ ^4^ ^]^

Early assessment of etiologic agents, such as viruses, is imperative for clinical point of care (POC). In medical diagnostics, virus recognition can be performed in laboratories using traditional methods, such as polymerized chain reaction (PCR) amplification and enzyme‐linked immunoassays, both of which require biological labels, such as radioisotopes, enzymes, and fluorophores, which can be readily detected using various analytical techniques. Although these methods are extremely sensitive and selective, it is common to employ multiple detection layers for several analytes. The need to engage highly trained staff, long analytical times, and the huge investment in effort and resources often hamper diagnosis. Considering the different diagnostic applications, there is an urgent need for effective virus sensors that are small and easy to operate and offer rapid response times as well as high selectivity, cross‐sensitivity, and portability.^[^ ^5^ ^]^

**Figure 3:**
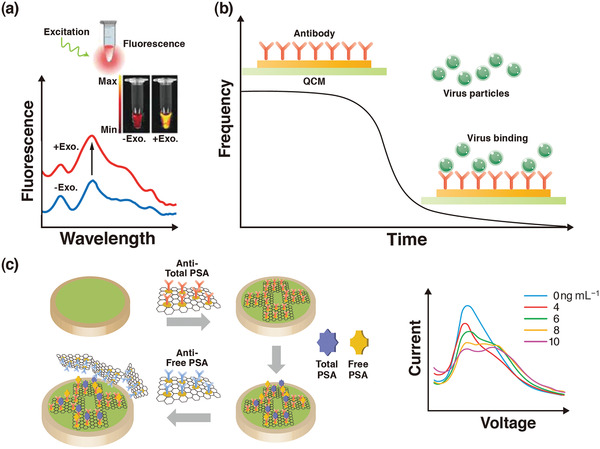
a) An overview of the main approaches using an optical biosensor. b) A piezoelectric biosensor. c) An electrochemical biosensor. a) Reproduced with permission.^[^ ^8^ ^]^ Copyright 2019, Wiley‐VCH. b) Reproduced under the terms of the CC‐BY Creative Commons Attribution 4.0 International license (https://creativecommons.org/licenses/by/4.0).^[^ ^5^ ^]^ Copyright 2017, The Authors, published by MDPI. c) Reproduced with permission.^[^ ^9^ ^]^ Copyright 2019, Wiley‐VCH.

Biosensors are described as compact analytical devices, incorporating biological or biomimetic sensing elements, and are applied for the detection and monitoring of various analytes or pathogens that are important for the environment, health, and food industries.^[^ ^6^ ^]^ Biosensors must meet the requirements of sensitivity, response accuracy, reproducibility, high specificity toward the desired target element, nontoxicity, and cost‐effectiveness.^[^ ^7^ ^]^ Biosensors can be divided into various groups, such as optical, piezoelectric, and electrochemical. **Figure** **3** presents an overview of the main approaches using transducers in biosensors. The simplest type of biosensor is an optical transducer,^[^ ^8^ ^]^ which can detect the analyte or pathogen as a measured change in the fluorescence, absorption, or reflectance performance of the sensing material. Figure 3a shows the fluorescence spectra of a semiconducting polyelectrolyte nanocomplex with and without exosomes (Exo.), as involved in pathogenesis‐including neurodegenerative diseases, viral/bacterial infections, and cancer. This technique simply takes advantage of the color change of the sensing materials when their size or concentration changes due to interaction with the analyte or pathogen. The second class of biosensors is the micromechanical transducer; the basis of this method is the measurement of changes in the resonance frequency.^[^ ^5^ ^]^ Quartz crystal microbalance (QCM) with piezoelectric properties, benefits from frequency changes (see Figure 3b) or deflection of the sensing material. The third class of biosensors is the electrochemical biosensor, which can be classified as amperometric, potentiometric, impedimetric, or conductometric.^[^ ^9^, ^10^ ^]^ Figure 3c shows a schematic illustration of an electrochemical sensor and the responses of square‐wave voltammetry of this sensor at concentrations of 0, 4, 6, 8, and 10 ng mL^−1^ of total prostate‐specific antigen (PSA). Graphene oxide/Au nanoparticles/antitotal PSA antibody is attached to the surface of the working electrode for antigen capture. The resulting antigen/antibody‐modified electrode is subsequently incubated with the prepared graphene oxide/Au nanoparticles/anti‐free PSA antibody to form a sandwich‐like system. The limit of detection of this biosensor is related to the large specific surface area of graphene oxide and the high electrochemical current of Au. It should be noted that in addition to the above three biosensors, other devices such as microfluidic,^[^ ^11^ ^]^ plasmonic,^[^ ^12^ ^]^ cantilever‐based^[^ ^13^, ^14^ ^]^ and field‐effect transistor (FET)‐based electronic^[^ ^15^, ^16^ ^]^ biosensors have been developed.

**Figure 4:**
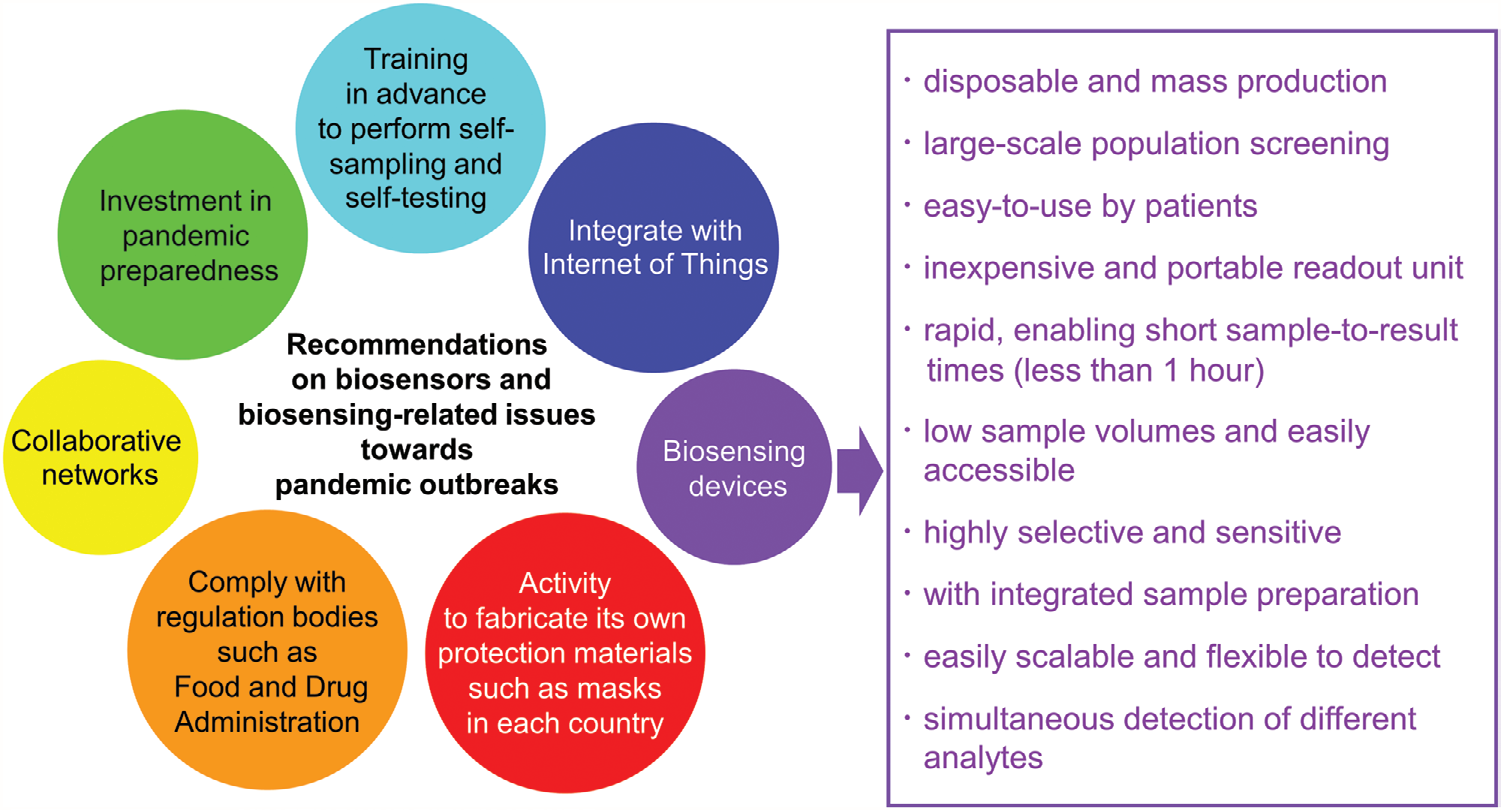
Seven main recommendations for the biosensing community and ten requirements for biosensing devices detecting infectious diseases.

Regarding the coronavirus disease 2019 (COVID‐19) pandemic that has never been experienced before in the 21st century, Ji et al.^[^ ^17^ ^]^ summarized the structure of SARS‐CoV‐2, its genomic and gene expression characteristics, the current progress of SARS‐CoV‐2 ribonucleic acid (RNA), antibodies, antigens, and virus detection. Morales‐Narváez and Dincer^[^ ^18^ ^]^ outlined innovative diagnostic methods for targeting a variety of COVID‐19‐related biomarkers. By measuring prognostic biomarkers and combining this knowledge with clinical observations and risk factors, patients can be stratified according to disease severity, so Russell et al.^[^ ^19^ ^]^ proposed the ideal features of the biosensors that can be regarded as a key player in precision medicine for COVID‐19. On the other hand, research and development of biosensors that can detect at an early stage with high sensitivity and low cost have been conducted. However, none of these are commercially available on the market, and cannot be used for pandemic diseases such as COVID‐19.^[^ ^20^ ^]^ Seven main recommendations have been suggested to the biosensing community (**Figure** **4**).^[^ ^18^, ^20^ ^]^ Recently, Samson et al.^[^ ^21^ ^]^ highlighted an overview of the traditional viral detection methods, and recent trends and future perspectives of biosensors for the detection of SARS‐CoV‐2.

Herein, the emphasis is on the piezoelectric materials since their coupled electromechanical properties^[^ ^22^ ^]^ make them well‐suited for use as sensors and actuators in smart structures and devices. In the past two decades, a wide range of piezoelectric ceramics and composites^[^ ^23^, ^24^ ^]^ have been studied by the Tohoku University research group. Piezoelectric materials have also become important biomaterials that can be interfaced with biological tissues and used in miniaturized bioelectronic and biochemical devices.^[^ ^25^ ^]^ Meanwhile, magnetostrictive materials can convert magnetic energy into mechanical energy or the reverse,^[^ ^26^, ^27^ ^]^ and are used to build sensors and actuators. In the past decade, works on magnetostrictive alloys and composites, including clad plates,^[^ ^28^ ^]^ short wire composites,^[^ ^29^ ^]^ and long wire composites,^[^ ^30^ ^]^ have been reported by our group. A new type of active magnetostrictive material has also been introduced as a biological sensor platform^[^ ^31^ ^]^ in recent years.

Piezoelectric ceramics/polymers and magnetostrictive alloys are examples of functional materials, which are promising energy‐harvesting materials.^[^ ^32^, ^33^ ^]^ Functional composite materials and energy‐harvesting technologies play an important role in building the Internet of Things (IoT) society.^[^ ^34^, ^35^, ^36^ ^]^ After the SARS‐CoV‐2 pandemic, environmental monitoring will become even more important. Also expected to gain importance is detection and monitoring of damage in composite materials^[^ ^37^, ^38^ ^]^ using electromagnetic sensors. Indeed, it is desirable to develop virus sensors that do not require power sources or batteries. Piezoelectric and magnetostrictive biosensors seem to exhibit superior performance to that of other biosensors (see **Table** **1**).^[^ ^39^, ^40^ ^]^ Optimal composite design will also ensure that the piezoelectric and magnetostrictive biosensors meet the requirements illustrated in Figure 4. However, further research progress is required; although the piezoelectric and magnetostrictive sensors can detect the viruses from frequency changes, these sensors have a potential to detect directly using the output voltage. In addition, they are expected to be combined with piezoelectric and magnetostrictive energy‐harvesting devices for the IoT, with the possibility to identify viruses by monitoring mechanical vibration. They can also be attached or embedded in smart clothing. Herein, we briefly discuss several human viruses with the main focus on piezoelectric and magnetostrictive materials of a layered configuration and energy‐harvesting capabilities, aiming to provide insights into the development of more sensitive virus sensors. Attention is drawn to the sensor material itself, whereas the receptor linked to the sensor that confers specificity to the detection is out of scope for the present review.

**Table 1:** Comparison of various detection approaches

Approach	Advantage	Disadvantage
Piezoelectric	Rapid Highly sensitive Specific Label‐free	Rely on sample preparation Complex pretreatment steps Brittleness
Magnetostrictive	Rapid Highly sensitive Specific Label‐free Simple configuration Wireless Easy fabrication	Pick‐up coil Eddy current (it limits the high frequency applications)
PCR	Sensitive Specific	Complex pretreatment steps Failing to distinguish between viable and nonviable cells Sophisticated instruments
Optical	Rapid Sensitive Specific	Complex pretreatment steps Sophisticated instruments
Electrochemical	Rapid Sensitive Label‐free Low cost	Low stability and repeatability Low coping ability for complex clinical samples

## 2 Piezoelectric Biosensors

**Figure 5:**
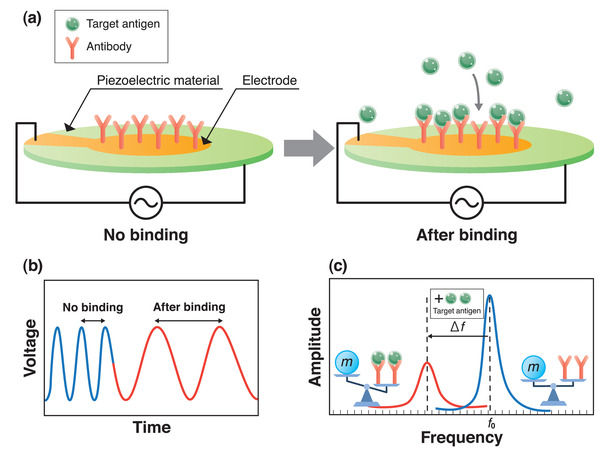
Basic concept of virus detection using piezoelectric material. a) Operation principle of a piezoelectric biosensor; b,c) schematics of voltage to time (b) and amplitude to frequency (c) during detection.

A piezoelectric material exhibits a mechanical oscillation under an alternating current (AC) voltage, producing an oscillating electric field. When a mass *m* increases due to the interactions between molecules, the frequency *f* controlled by the AC voltage decreases. Mass response‐type piezoelectric sensors are commonly used for virus detection. A schematic illustration of the operation principle of the piezoelectric biosensor is shown in **Figure** **5**. Probe antibodies are fixed on the upper electrode surface of the piezoelectric material as shown in Figure 5a. The upper and lower electrodes drive the resonation of the piezoelectric material. Target antigen then binds with the probe antibodies. The mass change Δ*m* on the electrode surface leads to a time‐dependent frequency shift (see Figure 5b) that is represented by the frequency change Δ*f* (Figure 5c) of the material in the oscillation circuit.^[^ ^41^ ^]^ Mainly anisotropic materials such as aluminum nitride (AlN),^[^ ^42^, ^43^, ^44^ ^]^ zinc oxide (ZnO),^[^ ^45^, ^46^ ^]^ barium titanate (BaTiO_3_),^[^ ^47^, ^48^, ^49^ ^]^ lead titanate (PbTiO_3_),^[^ ^50^, ^51^, ^52^ ^]^ quartz (SiO_2_),^[^ ^53^, ^54^ ^]^ and poly(vinylidene fluoride) (PVDF)^[^ ^55^, ^56^ ^]^ are used as sensor materials.^[^ ^57^ ^]^ **Table** **2** lists the Young's modulus *E*, shear modulus μ, Poisson's ratio ν, mass density ρ, longitudinal piezoelectric coefficient *d*_33_, and transverse piezoelectric coefficient *d*_31_ of each of these materials.

**Table 2:** Engineering constants of piezoelectric materials

	*E* [GPa]	μ [GPa]	ν	ρ [g cm^−3^]	*d*_33_ [pC N^−1^]	*d*_31_ [pC N^−1^]
AlN	308.3^[^ ^42^ ^]^	130.8^[^ ^42^ ^]^	0.179^[^ ^42^ ^]^	3.26^[^ ^43^ ^]^	6.72^[^ ^44^ ^]^	−2.71^[^ ^44^ ^]^
ZnO	112.2^[^ ^45^ ^]^	42.2^[^ ^45^ ^]^	0.336^[^ ^45^ ^]^	5.53^[^ ^45^ ^]^	12.3^[^ ^46^ ^]^	−5.12^[^ ^46^ ^]^
BaTiO_3_	112^[^ ^47^ ^]^	43^[^ ^48^ ^]^	0.35^[^ ^49^ ^]^	5.4^[^ ^47^ ^]^	140^[^ ^47^ ^]^	−60^[^ ^47^ ^]^
PbTiO_3_	213.7^[^ ^50^ ^]^	84.3^[^ ^50^ ^]^	0.26^[^ ^50^ ^]^	7.52^[^ ^51^ ^]^	79.1^[^ ^52^ ^]^	−23.1^[^ ^52^ ^]^
SiO_2_	72.52^[^ ^53^ ^]^	30.97^[^ ^53^ ^]^	0.166^[^ ^53^ ^]^	2.204^[^ ^53^ ^]^	*d*_11_ = 2.3^[^ ^54^ ^]^	*d*_14_ = −0.67^[^ ^54^ ^]^
PVDF	2^[^ ^55^ ^]^	0.752^[^ ^55^ ^]^	0.33^[^ ^55^ ^]^	1.8^[^ ^55^ ^]^	−22^[^ ^56^ ^]^	23^[^ ^55^ ^]^

**Figure 6:**
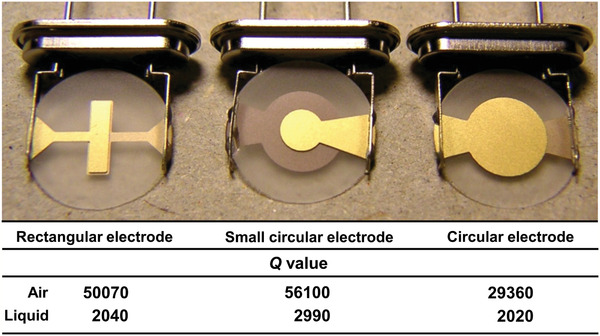
Photograph of quartz crystals and measured *Q* values; measurements were carried out in air and in phosphate‐buffered saline. Reproduced with permission.^[^ ^60^ ^]^ Copyright 2007, Elsevier.

Sauerbrey^[^ ^58^ ^]^ discovered the relation between the quartz oscillation frequency in thickness shear mode and change in surface mass. The Sauerbrey equation is given by Equation (1)

(1) $$\begin{array}{c} \Delta f\;\;=\;\;-\frac{2f_{0}^{2}}{A\sqrt{\rho \mu }}\Delta m \end{array}$$

where *f*_0_ is the fundamental resonance frequency and *A* is the active area of the piezoelectric material. The QCM technique, a label‐free technology, has made great progress^[^ ^59^ ^]^ and has been successfully applied in virus detection. QCM can exhibit extraordinary sensitivity to changes in resonance frequency, recording sub‐nanogram mass changes. The performance of the QCM is characterized using the mass sensitivity, which describes the shift in resonance frequency *f*_0_ due to adsorption of virus particles and the mechanical quality factor (*Q* value) defining the sharpness of the resonance peak. The *Q* value approximated as

(2) $$\begin{array}{c} Q\;\;=\;\;\frac{f_{0}}{\Delta s} \end{array}$$

where Δ*s* is defined as the spread of the signal trace at a distance from the baseline equal to the magnitude divided by $\sqrt{2}$. A higher *Q* value means a sharper resonance peak, resulting in a higher precision in determining resonance frequency and a smaller minimum detectable frequency change. The *Q* value can be optimized by the sensor electrode size and shape.^[^ ^60^ ^]^ **Figure** **6** shows three different AT‐cut 10 MHz quartz crystals together with the corresponding *Q* values in air and in a liquid environment.

Cantilever beams of microscale length and width and nanoscale thickness are also used as resonant sensors to detect a variety of biological and chemical entities.^[^ ^13^ ^]^ The resonance frequency of such a cantilever can be approximated by^[^ ^61^ ^]^

(3) $$\begin{array}{c} f_{0}\;\;=\;\;\frac{1}{2\pi }\sqrt{\frac{k}{m^{*}}} \end{array}$$

where m* = 0.24*ρlwh* and *k* = *Eh* ^3^ *w*/(4*l* ^3^) are the mass and spring constants of the cantilever, respectively, expressed as functions of the density ρ, Young's modulus *E*, length *l*, width *w*, and thickness *h*. The mass change of the beam in bending mode due to adsorption of virus particles is obtained by

(4) $$\begin{array}{c} \Delta m\;\;=\;\;\frac{k}{4\pi^{2}}\left[\frac{1}{{\left(f_{0}-\Delta f\right)}^{2}}\;-\;\frac{1}{f_{0}^{2}}\right] \end{array}$$

**Figure 7:**
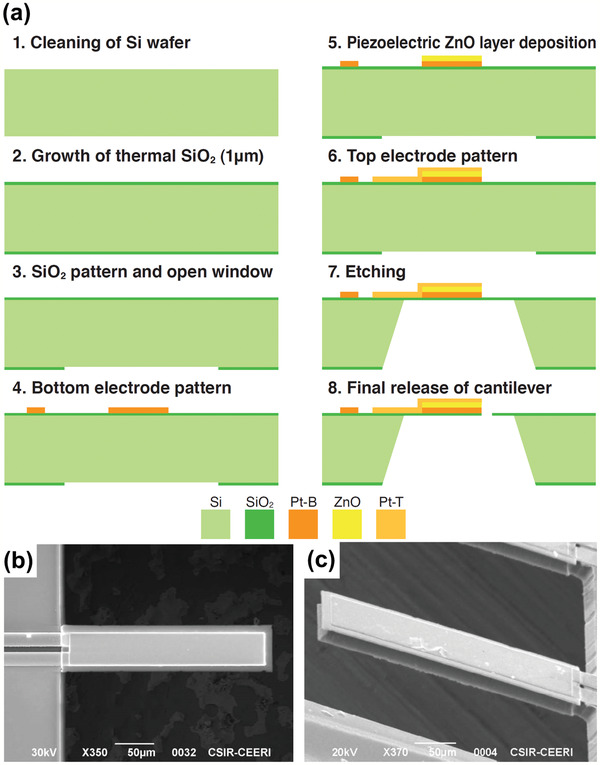
Microcantilever beam in piezoelectric biosensor. a) Fabrication process of a piezoelectric cantilever; b) top view of the cantilever; and c) cantilever coated with mass‐loading zinc oxide (ZnO) layer. a–c) Reproduced with permission.^[^ ^62^ ^]^ Copyright 2019, IEEE.

**Figure** **7**a shows an example of the fabrication process method for a microelectromechanical mass‐sensor based on the cantilever beam, actuated using a piezoelectric ZnO thin film.^[^ ^62^ ^]^ The beam stack consists of Si/SiO_2_/Pt/ZnO/Pt layers. First, Si wafers are cleaned and wafers are loaded in a thermal oxidation furnace for thermal SiO_2_ growth. The SiO_2_ layer is then patterned, and the Si windows are opened. The bottom Pt electrode (Pt‐B) is coated, and ZnO is deposited. After that, the top Pt electrode (Pt‐T) is coated, and the Si wafer is etched. Finally, the device structure is released, and the top window is opened. A selectively mass‐loading ZnO layer is deposited. Figure 7b,c shows the top view of bare and mass‐deposited cantilever beams, respectively.

### 2.1 Human Papilloma Virus: Group I

As the third most commonly occurring cancer in women, cervical cancer can be caused by human papillomavirus (HPV). HPV is a deoxyribonucleic acid (DNA) virus belonging to the Papillomaviridae family and is one of the most easily sexually transmittable viruses globally since millions of people have been infected with HPV at some point in their lives. It should also be noted that HPV viruses may have no effects in most people; however, some types can cause health problems such as genital warts or cancer. In February 2019, an HPV vaccine was introduced in 92 countries as part of a national vaccination program, but remains important to solve such problems as unstable vaccine supply, high delivery cost, and low seed rate still prevail. Given the prevalence of this virus, researchers focus on developing effective monitoring methods for high‐risk HPV communities.^[^ ^63^ ^]^

To identify the HPV in recurrent and original pathological biopsy samples, Fu et al.^[^ ^64^ ^]^ used AT‐cut 10 MHz piezoelectric quartz crystal and fabricated piezoelectric genesensors. A method for the rapid detection of HPV using piezoelectric genesensors was developed, and the detection effectiveness was compared with those of conventional PCR‐dot blot hybridizations. The researchers assumed that the results obtained from the piezoelectric genesensors and PCR‐dot blot were almost identical. Dell'Atti et al.^[^ ^65^ ^]^ combined DNA piezoelectric sensors with PCR to create label‐free DNA piezoelectric biosensors for detecting HPV from human cervical scraping specimens. They optimized the piezoelectric sensors with synthetic oligonucleotides and performed the tests on cervical scraping samples after PCR amplification. Reproducibility was expressed as the average coefficient of variation (CV%) for three samples, and a good reproducibility of approximately 10% was obtained. Chen et al.^[^ ^66^ ^]^ designed an adjustable, stainless‐steel, metal‐clamping, piezoelectric sensor. They detected the hybridization of HPV PCR products and discussed the effect of temperature change on the frequency baseline stability. Their results showed that the change in frequency amplitude can reach Δ*f* = 55 ± 7.4 Hz when the target product of 40 μL in an ice bath was added to 110 μL of the buffer. Prakrankamanant et al. combined the loop‐mediated isothermal amplification (LAMP) technique with QCM for real‐time detection of high‐risk HPV DNA type 58 (HPV‐58), most commonly found in Asian women.^[^ ^67^ ^]^ AT‐cut 9 MHz piezoelectric quartz crystals with polished Au electrode coatings on both sides were used. The effect of the changes in temperature and viscosity on the QCM sensor during the PCR process was addressed, and the sensitivity was further increased. The system could detect HPV‐58 at 100 copies with Δ*f* = 34 ± 3.6 Hz. However, the analyte in this method was in contact with the reusable sensor surface, which could lead to the risk of carryover contamination. It has also been reported that the diagnostic specificity is low (90.5%) because of disagreement in results of HPV‐58 positive by LAMP‐QCM but negative by the conventional LAMP technique. So, further research and development are required.

### 2.2 Vaccinia Virus: Group I

Vaccinia virus is the prototype virus of the orthopoxvirus genus in the family *Poxviridae* and is found in multiple infectious forms including intracellular mature virus.^[^ ^68^ ^]^ QCM technology was applied for real‐time airborne detection and quick detection of vaccinia virus.^[^ ^69^, ^70^ ^]^ Kleo et al.^[^ ^69^ ^]^ established a unique detection system for the identification of vaccinia virus by combining QCM detection techniques and PCR amplification. To ensure that the PCR products can be detected directly by the QCM‐based detection system, a pretreatment via denaturation (95 °C) and fast cooling down with precooled buffer (4 °C) was performed. The required analytical time for the new system was 15 min, which is less than those of the traditional methods. Lee et al.^[^ ^70^ ^]^ developed a method for the real‐time detection of airborne vaccinia virus using an integrated QCM technique. The capture rate varied linearly with the concentration of the initial virus suspensions (8.5 × 10^8^ to 8.5 × 10^10^ particles mL^−1^) at flow rates of 2.0 and 1.1 L min^−1^. The research demonstrated the potential of QCM on the detection of nanoscale biological entities in air.

### 2.3 Dengue Virus: Group IV

As a mosquito‐borne viral disease, dengue fever (DENV) has numerous patients.^[^ ^71^ ^]^ Although it is of major public health concern in urban and semiurban areas, leading to thousands of deaths per year, the current detection procedures are cumbersome and time‐consuming.^[^ ^72^, ^73^ ^]^ Wu et al.^[^ ^74^ ^]^ developed a dengue fever piezoelectric immunochip. They used a 10 MHz QCM consisting of an 8 mm AT quartz wafer placed between Au electrodes to detect dengue envelop protein (E protein) and nonstructural protein 1 (NS‐1 protein) in a viremia phase patient serum, and obtained a detection limit for the phosphate buffered saline (PBS) diluted samples. The immunochip failed to quantify dengue virus antigens in 1/1000 untreated samples, so they used the cibacron blue 3GA gel–heat denature (CB–HD) method and succeeded in reducing the dilution from thousandfold to hundredfold. They found that the CB–HD method was the most effective sample pretreatment technique, demonstrating detection limits as low as 1.727 µg mL^−1^ for E protein and 0.740 µg mL^−1^ for NS‐1 protein. Tai et al.^[^ ^75^ ^]^ coated the QCM with molecularly imprinted polymers (MIP) specific to the NS‐1 protein of flavivirus including dengue virus serotypes. The advantages of this MIP‐QCM to detect dengue virus were high sensitivity (1–10 ng mL^−1^), short operation time (20–30 min per sample), and easy interpretation.

Chen et al.^[^ ^76^ ^]^ established a circulating‐flow QCM biosensing method. Au nanoparticles (AuNPs) were integrated into AT‐cut 9 MHz piezoelectric quartz crystal. The specific oligonucleotide‐functionalized AuNP probes were used as both detection amplifiers and verifiers in this method. The temperature of the QCM system was maintained at 30 °C. The detection limit of the QCM instrument in liquid was 1 Hz, and the Δ*f* = 1 Hz corresponded to Δ*m* = 0.391 ng. In the nanoparticle application, this method was able to detect dengue virus cDNA in clinical blood samples at two plaque forming unit (PFU) per milliliter. A linear correlation (*R* ^2^ = 0.987) of the detection signal, i.e., Δ*f* versus virus titration, was found over a concentration range from 2 to 2 × 10^6^ PFU mL^−1^. Furthermore, the technique was label‐free and highly sensitive. Concentrations as low as 2 PFU mL^−1^ of DENV could be detected, compared to the 1–50 PFU mL^−1^ detection limit for fluorescent (i.e., not label‐free) real‐time PCR.

**Figure 8:**
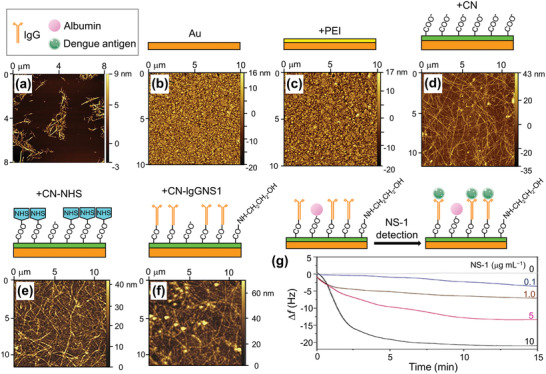
Piezoelectric immunochip coated with bacterial cellulose nanocrystals (CNs) for dengue virus detection. a) Atomic force microscopy (AFM) topography image of CN; b–d) AFM topography images of piezoelectric sensor surfaces coated with: b) Au, c) polyethylenimine (PEI) film, d) CN film; e,f) the interfaces obtained after *N*‐hydroxysuccinimide (HNS) activation (e) and monoclonal immunoglobulin G (IgGNS1) immobilization (f); and g) frequency shift profile during NS1 antigen (in blood serum diluted tenfold in phosphate buffered saline) recognition by the IgGNS1 using a quartz crystal microbalance with energy‐dissipation monitoring (QCM‐D). Before test recognizing the NS1 antigens, unspecific regions are blocked by injection of albumin solution. a–g) Reproduced with permission.^[^ ^78^ ^]^ Copyright 2017, Elsevier.

The commercialization of dengue virus sensors remains far from imminent due to remaining issues with clinical samples and the piezoelectric sensor itself.^[^ ^77^ ^]^ Pirich et al.^[^ ^78^ ^]^ proposed the functionalization of commercial piezoelectric sensors with cellulose nanocrystal (CN) thin films to anchor monoclonal immunoglobulin against NS‐1 dengue antigen. **Figure** **8**a shows the atomic force microscopy (AFM) topography image of CN, while Figure 8b–f shows the AFM topography images of piezoelectric sensor surfaces. Since the Au surface (Figure 8b) is known to hinder stable CN adsorption, a preliminary step was performed, coating the sensor with polyethylenimine (PEI) (see Figure 8c). The CN film was then anchored as shown in Figure 8d. The CN film was activated by an injection of 1‐ethyl‐3‐(3‐dimethylaminopropyl) carbodiimide/*N*‐hydroxysuccinimide (i.e., EDC/NHS) solution as shown in Figure 8e, and a monoclonal immunoglobulin G (IgGNS1) solution was injected for immobilization (Figure 8f). QCM was used to assess both antigen recognition and the frequency change Δ*f* of the immunochips during the assembly process. Interaction analyses of the activation of the CN thin films were also performed by a QCM with energy‐dissipation monitoring (QCM‐D). The system was found capable of detecting IgGNS1 in the range of 0.01–10 µg mL^−1^ by both QCM and QCM‐D. The detection limits were 0.32 µg mL^−1^ for QCM and 0.1 µg mL^−1^ for QCM‐D. Figure 8g shows the frequency change Δ*f* over time during NS‐1 detection using the QCM‐D. QCM and QCM‐D apparatuses can be employed in NS‐1 recognition and show potential for more sensitive, faster, and/or less expensive diagnostic assays for dengue (**Table** **3**).

**Table 3:** Reported limits of detection for dengue virus

Target	Detection limit	Ref.
E protein	1.727 µg mL^−1^	^[^ ^74^ ^]^
NS‐1	0.740 µg mL^−1^	^[^ ^74^ ^]^
NS‐1	1–10 ng mL^−1^	^[^ ^75^ ^]^
cDNA	2 PFU mL^−1^	^[^ ^76^ ^]^
NS‐1	0.1 µg mL^−1^	^[^ ^78^ ^]^

### 2.4 Ebola Virus: Group V

Ebola virus was first identified in 1976, and thousands of people, including numerous health care workers, have died due to this disease.^[^ ^79^ ^]^ Ebola virus disease (EVD), formerly known as Ebola hemorrhagic fever, is a severe, often fatal illness affecting humans and other primates. The virus is transmitted to people from wild animals (such as fruit bats, porcupines, and nonhuman primates) and then spreads in the human population through direct contact with the blood, secretions, organs, or other bodily fluids of infected people, and with surfaces and materials (e.g., bedding and clothing) contaminated with these fluids. The average EVD case fatality rate is around 50%. Case fatality rates varied from 25% to 90% in past outbreaks.^[^ ^80^ ^]^ Rapid POC care detection of the Ebola virus could enable early quarantine and help halt pandemics.

Baca et al.^[^ ^81^ ^]^ fabricated a surface acoustic wave (SAW) sensor and proposed a label‐free sensing system for the rapid detection of Ebola antigens at the POC without the need for added reagents, sample processing, or specialized personnel. The sensor chips were prepared using lithium tantalate (LiTaO_3_) wafers with inter‐digital transducers (IDTs). The piezoelectric substrate propagated horizontally polarized surface shear waves induced by application of an AC voltage (several hundred millivolts to several volts) to the IDTs at a high frequency between 80 and 400 MHz. Molecular interactions between virus and antibody emitted an acoustic wave leading to a change in the input frequency. That is, detection of Ebola virus resulted in a frequency increase, with phase shift values ranging from 0.20 ± 0.04° to 4.46 ± 0.86°. They observed a log–linear sensor response for Ebola viral particles, with a detection limit of 1.9 × 10^4^ PFU mL^−1^. They predicted that the SAW sensor would greatly improve testing sensitivity for infectious Ebola virus. The SAW sensor phase shift response seems to perform well for an infectious Ebola sample. However, the characteristics of acoustic waves depend on the temperature, so attention must be paid to the measurement environment.

### 2.5 Influenza A Virus: Group V

New viruses are causing outbreaks, and old viruses grow stronger every day, influenza virus (types A, B, C, and D) among them. Hence, current sensing methods require continuous upgrading to manage the numerous growing challenges for virus diagnosis.^[^ ^57^ ^]^ Influenza A virus, a general type, has high mutagenicity and infectivity and is the most prevalent and severe infection. Type A exhibits a route of transmission from person to person as well as from animal (bird, pig, horse, etc.) to person. Influenza B virus has a slower rate of change than type A and has relatively mild symptoms. Although infectivity is high, the infection route is from person to person, and it is less likely than type A to cause a high fever. Once influenza C viral immunity has been acquired, that immunity persists for life. Reinfection is reminiscent of the common cold. The influenza D is suggested to be one of the viruses responsible for bovine respiratory disease complex.

**Figure 9:**
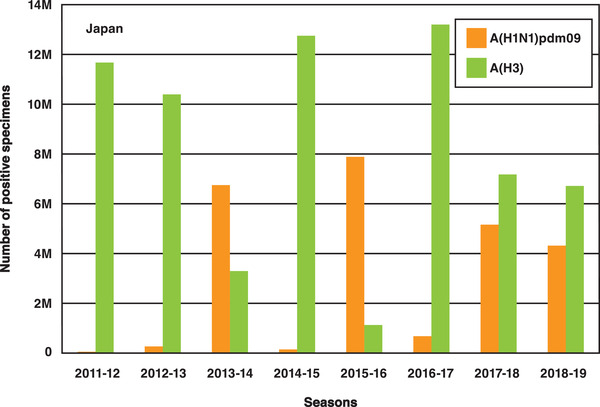
Number of specimens positive for influenza A in Japan in the period 2011–2019.

The influenza A virus is often discussed as a worldwide influenza epidemic. As the shape of the virus continues to evolve, acquired immunity performance drops, and strain prediction becomes more difficult for vaccine producers. **Figure** **9** shows changes in the number of people infected with the influenza A virus in Japan over the past 10 years. According to the World Health Organization, the A(H1N1)pdm09 strain, which is a new version of the H1N1 (commonly known as the Spanish flu), was responsible for the 2009 influenza pandemic. A part of H3 is known as the Hong Kong Cold. Since current methods for the diagnosis of influenza require specialized laboratory facilities and highly trained personnel and, in the case of viral culture, can take up to 14 days to obtain a definitive result, a QCM‐based sensor has been developed for the rapid detection of both influenza A and B viruses in laboratory‐cultured preparations and clinical samples.^[^ ^82^ ^]^

**Figure 10:**
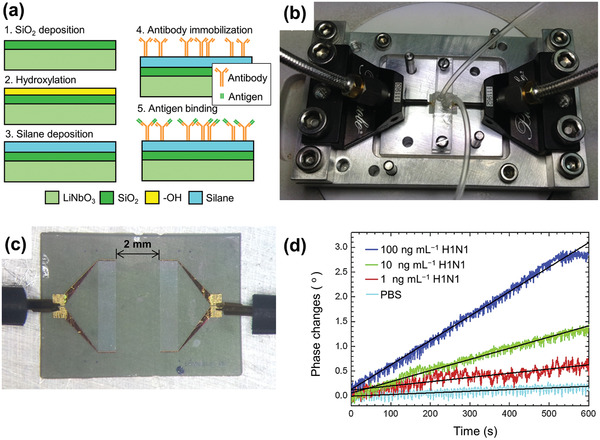
Surface acoustic wave (SAW) sensor for influenza A virus detection. a) Preparation of bioactive surfaces for SAW sensors; b) photograph of a SAW sensor mounted onto a fixture with low‐loss microwave probes; c) photomicroscopy image of the microwave probes in contact with the electrodes; and d) phase change versus time for a SAW sensor exposed to H1N1 HA antigen solutions of various concentrations and phosphate‐buffered saline (PBS). a–d) Reproduced with permission.^[^ ^83^ ^]^ Copyright 2015, Elsevier.

**Figure 11:**
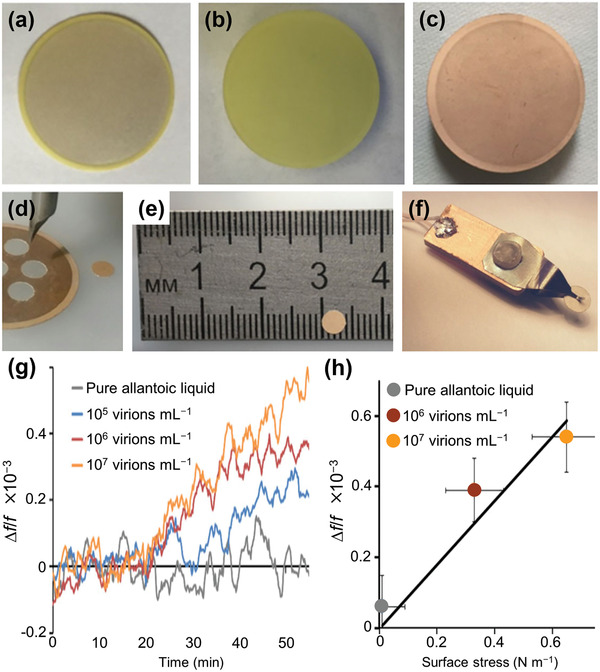
Disk sensor for influenza A virus detection. a–f) Fabrication process: a) Ag‐coated lead zirconate titanate (PZT) plate of 100 µm thickness; b) plate after incubation in 98% nitric acid solution; c) Au‐coated plate (coating thickness: 50 nm); d,e) 4 mm disk cut with chopped carbide grade from laminated Au‐coated plate; f) holder that clamps disk in the center. g) Resonance frequency shift as a function of time for different virus concentrations; and h) frequency shift as a function of surface stress due to virus concentrations. a–h) Reproduced under the terms of the CC‐BY Creative Commons Attribution 4.0 International license (https://creativecommons.org/licenses/by/4.0).^[^ ^84^ ^]^ Copyright 2019, The Authors, published by The Royal Society.

Jiang et al.^[^ ^83^ ^]^ designed and fabricated Love wave SAW sensors with SiO_2_‐coated lithium niobate (LiNbO_3_) piezoelectric wafers for the detection of influenza A viral antigen. **Figure** **10**a shows a schematic of the preparation of the active surface for the SAW sensors. Figure 10b,c shows the test setup. Figure 10d shows the phase changes measured for the SAW sensor exposed to PBS powder and H1N1 HA antigen solutions. A detection limit as low as 1 ng mL^−1^ was obtained for influenza A H1N1 HA antigen at room temperature. Erofeev et al.^[^ ^84^ ^]^ described label‐free rapid detection of influenza A virus using Ag‐coated lead zirconate titanate (PZT) piezoelectric disks of 100 µm thickness. The disks, modified with synthetic sialylglycopolymers based on a polymer matrix, which biospecifically bind the haemagglutinin proteins on the influenza viruses, were inserted in a flowing virus suspension. **Figure** **11**a–f shows the fabrication process of the PZT disk sensor. Label‐free detection of the virus was achieved by monitoring the shift in disk radial mode resonance frequency. Figure 11g presents differences between the resonance frequency shifts for influenza A virus concentrations of 0, 10^5^, 10^6^, and 10^7^ virions mL^−1^. Label‐free detection of influenza A viruses at concentrations below 10^5^ virions mL^−1^ was demonstrated. It was also shown that frequency shift is proportional to the surface stress induced by virus adsorption (Figure 11h). Furthermore, the sensitivity was found to be inversely proportional to the thickness of the resonator. Hence, by using a thinner PZT substrate, the sensitivity can easily be increased several‐fold. It is expected that this PZT disk sensor method for influenza A virus detection can be extended to home application.

### 2.6 Human Immunodeficiency Virus: Group VI

HIV was first documented as a dangerous blood‐borne pathogen in the early 1980s, and HIV detection methods have gradually improved in terms of both sensitivity and specificity.^[^ ^85^ ^]^ HIV is a lentivirus that leads to acquired immunodeficiency syndrome (AIDS), and with increasing awareness of AIDS emerging as a global public health threat, a wide range of biosensors have been developed for early diagnosis of HIV infections.^[^ ^86^ ^]^

Properties of piezoelectric acoustic sensors using AT‐cut 20 MHz quartz wafer have been evaluated for HIV‐1 (nonspecific) versus HIV‐2 (specific) antibody–antigen interactions.^[^ ^87^ ^]^ It has been shown that the introduced sensor is effective in detecting and distinguishing HIV‐2 from HIV‐1 antibodies with good selectivity. Lu et al.^[^ ^88^ ^]^ fabricated a MIP‐coated QCM biomimetic sensor for the detection of HIV‐1‐related protein (glycoprotein 41(gp‐41)). It was found that the MIP film not only exhibited a strong affinity for the template peptide, but also could specifically bind the corresponding HIV‐1 protein. The detection limit was 2 ng mL^−1^.

**Figure 12:**
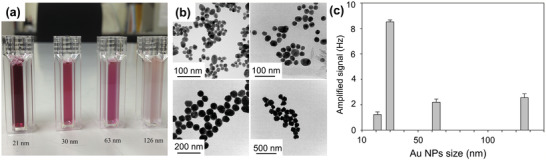
Quartz crystal microbalance (QCM) for human immunodeficiency virus (HIV)‐1 detection. a) Au nanoparticle (AuNP) solutions; b) TEM images of Au particles; and c) frequency changes upon streptavidin–Au immobilization. The error bars represent the standard deviations of four replicates. a–c) Reproduced with permission.^[^ ^90^ ^]^ Copyright 2016, Elsevier.

PCR and antibody capture by an enzyme‐linked immunosorbent assay for HIV‐1 and HIV‐2 are time‐consuming and require sophisticated equipment that is not compatible with emergency POC requirements. Bisoffi et al.^[^ ^89^ ^]^ developed a prototype biosensor based on functionalized piezoelectric materials with specific antibodies against HIV‐1 and HIV‐2. They prepared a new generation of biosensor chips with altered dimensions and an increased area occupied by the IDTs as wafers. They employed lithographic deposition and patterning of the IDT and the SiO_2_ waveguide layers, followed by cutting of the final format of the chips. While most other technologies require 30–60 min to detect a potential infection, the developed biosensor detected the presence of virus within 5 min, including the distinction between HIV‐1 and HIV‐2. Hence, this prototype biosensor may have the potential to be developed into a device for use in applications that require rapid and reliable testing for potential HIV infections in blood donors. HIV‐1 antigens were detected by the QCM using AuNPs as a signal enhancer.^[^ ^90^ ^]^ The QCM sensor, consisting of cavity resonators constructed over a piezoelectric substrate, would accumulate electric charges in response to the applied stress. Four AuNPs of different sizes were prepared (see **Figure** **12**a). Figure 12b shows the transmission electron microscopy (TEM) image of the resulting AuNPs, with average diameters of 21, 30, 63, and 126 nm. Figure 12c shows the effect of NP size on the detection signal. The target HIV‐1 antigen concentration was 1 ng mL^−1^. Streptavidin–Au with a size of 30 nm yielded the strongest signal. That is, using streptavidin–Au as an amplifier, a limit of detection of 1 ng mL^−1^ was reached.

### 2.7 Hepatitis B Virus: Group VII

**Figure 13:**
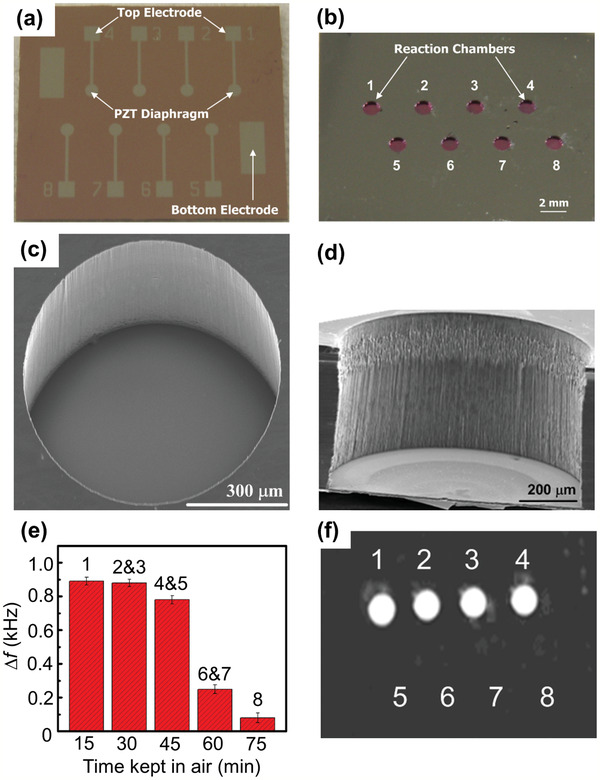
Micro‐piezoelectric immunoassay chip for hepatitis B virus detection. a,b) Optical image of front‐side (a) and reverse‐side (b) views of a piezoelectric sensor array. c,d) Top (c) and cross‐sectional (d) SEM image views of a reaction chamber. e) Measured frequency shift due to the binding activity of the immobilized antigen. The error bars represent the standard deviations of three replicates. f) Fluorescence image of the piezoelectric sensor array after capturing the specific antibodies. a–f) Reproduced with permission.^[^ ^92^ ^]^ Copyright 2011, Elsevier.

Hepatitis B virus (HBV) infection is one of the most common health problems in the world.^[^ ^91^ ^]^ Although billions of people suffer from HBV, effective medicine and treatment are unfortunately not found to cure chronic HBV infection. Therefore, detecting and monitoring HBV during the early stages of infection is of great importance. Xu et al.^[^ ^92^ ^]^ developed a piezoelectric diaphragm‐based immunoassay chip using microfabrication technology to detect anti‐HBV and anti‐alpha‐fetoprotein (AFP). Firstly, a thin TiO_2_/Pt film was sputter‐deposited on the top side of a Si‐on‐insulator wafer as the bottom electrode. A thin PZT film was then deposited by a sol–gel deposition method, and the PZT film was wet‐etched. A Si_3_N_4_ layer was deposited, and the Ti/Pt top electrode was sputtered. **Figure** **13** shows optical and scanning electron microscopy (SEM) images of the fabricated sensor chip. The chip consists of eight individual sensors in a sandwich structure with a circular top electrode, a PZT diaphragm, and a bottom electrode, as shown in Figure 13a. Figure 13b presents the back‐side of the sensor array. Figure 13c shows a top‐view SEM image of one reaction chamber, while Figure 13d shows a cross‐sectional SEM view of the same. Figure 13e shows the processing time effect on the frequency change due to the binding activity of the antigen. For example, sensors 2 and 3 were kept in air for 30 min before applying antigen solution, and the frequency was measured after the interaction time. Sensors 1 to 4 successfully captured the antibodies, as shown in Figure 13f. A detection limit of 0.1 ng mL^−1^ was obtained from the frequency shift‐based calibration curves. The fabricated sensor chip could be used to simultaneously detect multiple analytes. Giamblanco et al.^[^ ^93^ ^]^ described a single‐step, label‐free method to selectively detect the HBV genome. Au‐coated QCM crystals consisting of a Au layer with a chromium adhesion layer were used, and QCM‐D measurement was carried out. At a probe density of 4.0 × 10^12^ molecules cm^−2^, a sensitivity of tens of ng cm^−2^ could be obtained for the HBV target without using any amplification steps or labeling method.

**Figure 14:**
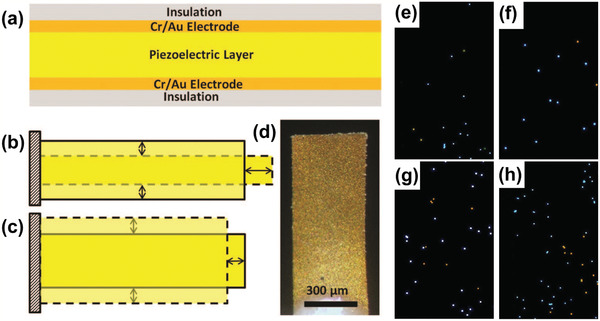
a) Schematic of a piezoelectric plate sensor for Hepatitis B virus double mutation (HBVDM) detection; b) the first length‐extension mode and c) width‐extension mode vibration of the piezoelectric plate sensor, with the shaded bars indicating the initial position of the piezoelectric plate sensor and the dashed shapes illustrating the extended positions. d) Top‐view optical microscopy image and e–h) fluorescence images of the sensor obtained after fluorescent reporter microsphere detection that followed the mutation detection in a mixture of mutant with 250 times more wild type at mutation concentrations of: e) 100 × 10^−21^ m, f) 1 × 10^−18^ m, g) 10 × 10^−18^ m, and h) 100 × 10^−18^ m. a–h) Reproduced with permission.^[^ ^94^ ^]^ Copyright 2015, The Royal Society of Chemistry.

To further improve detection performance, piezoelectric plate technology should be combined with other technologies such as sensitive membranes, microfluidics, and nanoparticles. By using a (Pb(Mg_1/3_Nb_2/3_)O_3_)_0.65_(PbTiO_3_)_0.35_ (i.e., PMN–PT) piezoelectric plate sensor coated with probe DNA, in situ detection of HBV double mutation (HBVDM) in urine has been previously discussed.^[^ ^94^ ^]^ The PMN–PT layer coated with Au electrodes on the two major surfaces and encapsulated by thin electrical insulation is shown in **Figure** **14**a. The binding of the target DNA from the biological fluid sample to the probe DNA on the piezoelectric plate sensor surface shifted the sensor length‐extension mode (Figure 14b) and width‐extension mode (Figure 14c) resonance frequencies. Figure 14d shows an optical microscopy image of the sensor. This piezoelectric plate sensor was shown to detect HBVDM with an analytical sensitivity of 60 copies mL^−1^. After the test, the sensor was examined using a fluorescence microscope, and the obtained fluorescence images from detection at mutation concentrations of 100 × 10^−21^ m (10^−16^ mol m^−3^), 1 × 10^−18^ m (10^−15^ mol m^−3^), 10 × 10^−18^ m, and 100 × 10^−18^ m are shown in Figure 14e–h, respectively. The blue and orange spots represent the mutation fluorescent reporter microspheres and wild type fluorescent reporter microspheres. The PMN–PT piezoelectric plate sensor was also able to detect double‐stranded HBVDM and K‐ras point mutation with a detection efficiency of 70% or better at concentrations as low as 10^−19^ m (10^−16^ mol m^−3^) against single‐stranded mutation detection at the same concentrations.^[^ ^95^ ^]^

## 3 Magnetostrictive Biosensors

**Figure 15:**
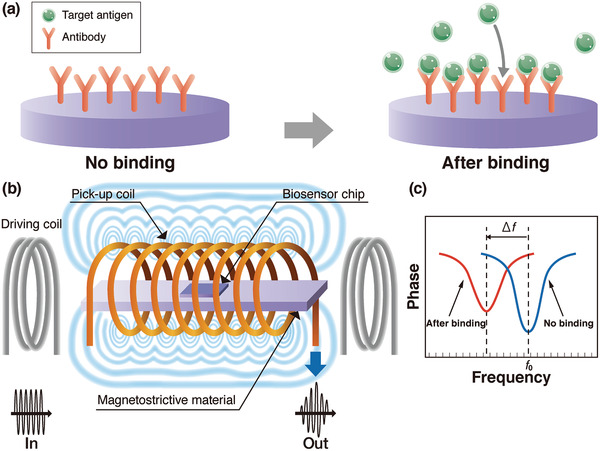
Basic concept of virus detection using magnetostrictive material. a) Operation principle of a magnetostrictive biosensor; b) schematic of magnetostrictive microcantilever (MSMC), driving coil and pick‐up coil; and c) schematic of amplitude to frequency during detection.

The magnetostrictive microcantilever (MSMC) has been investigated as a remote biosensor platform^[^ ^96^ ^]^ that works well in either air or liquid. The principle of the biosensor is based on the resonance frequency change Δ*f* with the mechanical load change as described in Section 2. A schematic illustration of the operation principle of the MSMC is shown in **Figure** **15**. Probe antibodies are fixed on the upper biosensor chip as shown in Figure 15a. Due to the magnetostrictive effect, the application of an AC magnetic field using a driving coil as shown in Figure 15b induces an oscillation of the MSMC. The attachment of a mass load Δ*m* such as an antigen to the sensor surface with probe antibodies lowers the resonance frequency (see Figure 15c). The oscillation results in an emission of a magnetic flux, and changes in the amplitude and phase signal of the oscillation lead to a magnetic flux change that can be detected using a pick‐up coil (Figure 15b). Metglas amorphous alloy,^[^ ^97^, ^98^, ^99^ ^]^ Fe–Co alloy,^[^ ^32^, ^100^, ^101^ ^]^ and cobalt ferrite (CoFe_2_O_4_) ceramics^[^ ^102^, ^103^, ^104^ ^]^ are widespread materials for magnetostrictive applications. **Table** **4** lists the Young's modulus *E*, Poisson's ratio ν, mass density ρ, piezomagnetic constant $d_{33}^{m}$, and magnetostriction λ values for these materials.

**Table 4:** Engineering constants of magnetostrictive materials

	*E* [GPa]	ν	*ρ *[g cm^−3^]	$d_{33}^{m}$ [nm A^−1^]	λ [ppm]
Metglas	100^[^ ^97^ ^]^	0.33^[^ ^98^ ^]^	7.9^[^ ^97^ ^]^	50.3^[^ ^99^ ^]^	11^[^ ^97^ ^]^
Fe–Co	182^[^ ^100^ ^]^	0.3^[^ ^100^ ^]^	8.4^[^ ^32^ ^]^	0.125^[^ ^101^ ^]^	80–140^[^ ^32^ ^]^
CoFe_2_O_4_	154^[^ ^102^ ^]^	0.37^[^ ^102^ ^]^	5.29^[^ ^103^ ^]^	−1.88^[^ ^102^ ^]^	−273^[^ ^104^ ^]^

MSMC performance is characterized by the mass sensitivity and the *Q* value. For MSMCs made of a commercial magnetostrictive Metglas alloy, for example, the *Q* value can reach more than 500 when operated in air and 30 when operated in water.^[^ ^96^ ^]^ In addition, the *Q* value of Metglas under multiharmonic resonance modes can be as high as 1000 in air and over 100 in water.^[^ ^105^ ^]^ On the other hand, magnetoelastic particles composed of an amorphous iron–boron binary alloy exhibit a *Q* value of 656 in air.^[^ ^106^ ^]^ Compared to piezoelectric microcantilevers, MSMCs have simple configurations.

For a thin sensor vibrating in its basal plane, the fundamental resonant frequency of the longitudinal vibrations is given by^[^ ^98^ ^]^

(5) $$\begin{array}{c} f_{0}\;\;=\;\;\frac{1}{2l}\sqrt{\frac{E}{\rho (1-\nu^{2})}} \end{array}$$

If the mass increase is small compared to the initial mass *ρlwh* of the sensor, then the mass change of the plate in longitudinal mode due to adsorption of virus particles, according to the measured resonance frequency shift, is given by^[^ ^107^ ^]^

(6) $$\begin{array}{c} \Delta m\;\;=\;\;-2\rho lwh\frac{\Delta f}{f_{0}} \end{array}$$

Equation (6) shows that increasing mass on the sensor surface produces a linear reduction in resonance frequency. If the mass of a single virus is known in advance, the number or type of the virus can be identified from the change in frequency.

From Equation (6), it can be seen that the sensitivity (Δ*f*/Δ*m*) is proportional to the resonance frequency *f*_0_ and inversely proportional to the magnetostrictive biosensor mass *ρlwh*. This feature does not apply to the piezoelectric biosensors (see Equation (1) or (4)). Smaller magnetostrictive biosensors have higher *f*_0_ and lower *ρlwh*, leading to higher sensitivity. However, as the biosensor size decreases, the amplitude of the detected signal decreases and the signal‐to‐noise ratio decreases, making signal processing harder.^[^ ^108^ ^]^ Theoretical results showed that the sensitivity depends on the mass distribution^[^ ^109^ ^]^ and is proportional to the square of mode shape^[^ ^110^ ^]^ of the sensor.

MSMC shows numerous advantages: 1) its actuation and sensing unit is wirelessly controlled; 2) its fabrication process is relatively easy; and 3) it works well in liquids.^[^ ^31^ ^]^

### 3.1 Bacterial Spore

The magnetostrictive platform has a unique advantage over conventional sensor platforms in that measurement is wireless and remote. This advantage is achieved by using the driving and pick‐up coils, as shown in Figure 15b.

A biosensor for the detection of bacterial spores has already been developed. Metglas 2826 MB alloy was used as the sensor platform. The composition is Fe_40_Ni_38_Mo_4_B_18_, with a theoretical saturation magnetostriction value of 11 ppm. The detection targets were yeast cell^[^ ^96^ ^]^ and acid phosphatase.^[^ ^111^, ^112^ ^]^ This sensor was also used to detect *Salmonella typhimurium* bacteria. Guntupalli et al.^[^ ^113^ ^]^ constructed a biosensor immobilizing a polyclonal antibody onto the surface of Metglas 2826 MB alloy, in order to detect *S. typhimurium* in air. Because the increased mass of *S. typhimurium* was very small compared to the initial mass of the biosensor, they determined the mass change due to the bacteria binding from Equation (6). They compared the density of bacteria cells calculated from Equation (6) and the density of bacteria cells measured by SEM, and obtained good agreement between the different methods. Detection limit of 5 × 10^3^ colony forming unit (CFU) mL^−1^ was obtained for 15 µm thick sensor with size of 2 mm × 0.4 mm. Fu et al.^[^ ^114^ ^]^ demonstrated the detection of *S. typhimurium* bacteria in water using the Cu/Metglas bilayer.

Johnson et al.^[^ ^106^ ^]^ fabricated magnetoelastic particles composed of an amorphous iron–boron binary alloy Fe_79_B_21_ by sputtering onto a chromium and Au‐coated Si wafer, and explored functionality in the detection of *Bacillus anthracis* spores. They obtained a correlation between the actual number of spores bounded to the biosensor and the calculated mass increase based on the resonance frequency shift from the experiments. Li et al.^[^ ^115^ ^]^ performed the in situ detection of *B. anthracis* spores in water in a real‐time manner using a Cu/Metglas bilayer. Li and Cheng^[^ ^105^ ^]^ then showed that the sensitivity of the biosensor is strongly dependent on the location of the mass load. The detection of *Escherichia coli* ^[^ ^116^ ^]^ and *B. Anthracis* spores^[^ ^117^ ^]^ in water has been reported using the Cu/Metglas bilayer. Biosensors for in situ detection of pathogenic bacteria in liquid has been developed using magnetostrictive Metglas particles.^[^ ^39^, ^118^ ^]^ *S. typhimurium*, *Listeria monocytogenes*, *E. coli*, and *Staphylococcus aureus* were characterized. In addition, Metglas 2826 MB alloy biosensors specific to *S. typhimurium* have been prepared by immobilizing antibody or phage as biorecognition elements onto the sensor.^[^ ^119^ ^]^ It was demonstrated that bacteriophage immobilized sensors have much better thermal stability than antibody immobilized sensors. **Table** **5** summarizes the detection limit of the Metglas sensor as applied to various targets.^[^ ^113^, ^114^, ^115^, ^116^, ^117^, ^118^, ^119^, ^120^ ^]^

**Table 5:** Reported limits of detection for various harmful substances

Target	Detection limit	Ref.
*S. typhimurium*	5 × 10^3^ CFU mL^−1^	^[^ ^113^ ^]^
*B. anthracis* in water	10^5^ CFU mL^−1^	^[^ ^115^ ^]^
*E. coli* in water	10^5^ CFU mL^−1^	^[^ ^116^ ^]^
*B. anthracis* in water	10^4^ spores mL^−1^	^[^ ^117^ ^]^
Pathogens in water	100 CFU mL^−1^	^[^ ^118^ ^]^
Octachlorostyrene	2.8 × 10^−9^ m	^[^ ^120^ ^]^
Human serum albumin	0.039 µg mL^−1^	^[^ ^121^ ^]^
Carcinoembryonic antigen	1 pg mL^−1^	^[^ ^122^ ^]^

### 3.2 Protein

**Figure 16:**
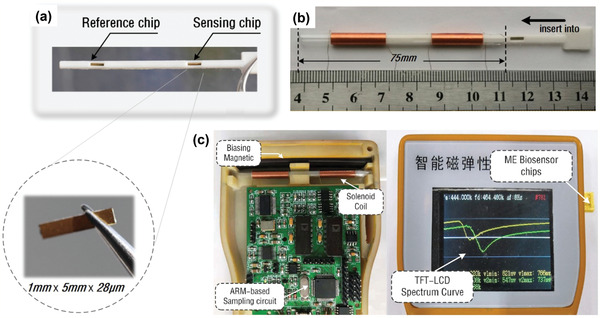
Magnetostrictive sensor for human serum albumin detection. a) Two test slots for magnetostrictive biosensor chips including reference and sensing chips; b) components and assembly process of magnetostrictive biosensor; and c) components of the sensing system. a–c) Reproduced with permission.^[^ ^121^ ^]^ Copyright 2019, Elsevier.

The Metglas 2826 MB biosensor for the detection of glucose^[^ ^108^ ^]^ has been developed. Recently, Sang et al.^[^ ^121^ ^]^ designed small, cost‐effective, stable Metglas 2826 MB biosensor to detect human serum albumin (HSA) rapidly and specifically, with a detection limit as low as 0.039 µg mL^−1^ (Table 5). **Figure** **16** shows the magnetostrictive sensor and sensing system. Wang et al.^[^ ^122^ ^]^ developed the Metglas nano‐biosensor for detecting carcinoembryonic antigen (CEA) by biological modification on the surface of the sensor platform. The nano‐biosensor had a linear response to the logarithmic CEA concentrations ranging from 2 pg mL^−1^ to 6.25 ng mL^−1^, with a detection limit of 1 pg mL^−1^ (Table 5) and a sensitivity of 105.05 Hz mL ng^−1^.

### 3.3 Classical Swine Fever: Group IV

**Figure 17:**
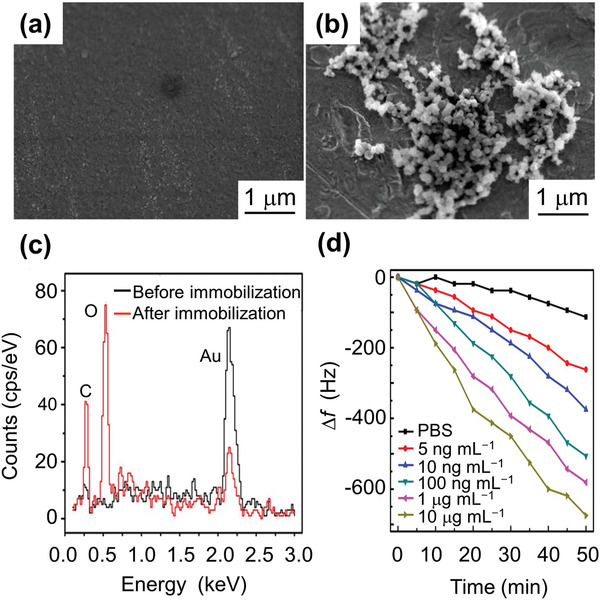
Magnetostrictive sensor for classical swine fever virus (CSFV) detection. a) SEM image of the Au‐coated sensor surface; b) SEM image of the biosensor surface after CSFV E2 immobilization; and c) EDS spectrum of the biosensor surface before and after the CSFV E2 immobilization. After CSFV E2 immobilization, it is clear that the Au content is reduced while the carbon and oxygen accumulations are dramatically increased. E2 contains large amounts of carbon and oxygen due to the nature of the envelope glycoprotein. d) Frequency shift as a function of time for phosphate buffered saline (PBS) and different anti‐CSFV E2 antibody concentrations. a–c) Reproduced with permission.^[^ ^125^ ^]^ Copyright 2017, Springer Nature. d) Reproduced with permission.^[^ ^124^ ^]^ Copyright 2016, Elsevier.

Classical swine fever, which is caused by the swine fever virus (CSFV), is a serious, economically damaging disease of swine,^[^ ^123^ ^]^ and CSFV detection has been extensively investigated in connection with economic damage to the pig industry. A magnetostrictive sensing system for the detection of CSFV has been published.^[^ ^124^ ^]^ In that study, the magnetostrictive sensor platform was composed of Metglas 2826 alloy (Fe_40_Ni_40_P_14_B_6_). The experimental data showed a sensitivity of approximately 95 Hz mL µg^−1^ for the CSFV detection sensor, with a detection limit of 0.6 µg mL^−1^. A magnetostrictive sensor immobilized with E2 glycoprotein was developed to detect CSFV E2 antibodies.^[^ ^125^ ^]^ **Figure** **17**a–c shows a SEM image of the sensor's Au surface without and with functionalization, as well as the energy‐dispersive X‐ray spectroscopy (EDS) spectra for elemental analysis of the sensor surface before and after the immobilization of CSFV E2. It was found that the Au content decreases after CSFV E2 immobilization. On the other hand, E2 contains large quantities of carbon and oxygen due to the nature of the envelope glycoprotein, so it is clear that carbon and oxygen accumulation also increased dramatically after CSFV E2 immobilization. From Figure 17d, it was observed that the resonance frequency shift increases with increasing CSFV E2 antibody concentration. The sensor showed a linear response to the logarithm of CSFV E2 antibody concentration, with a sensitivity of 56.2 Hz mL µg^−1^ and a detection limit of 2.466 ng mL^−1^. This sensor constituted a low cost, high sensitivity, wireless method for the selective detection of CSFV E2 antibodies. This CSFV E2 study not only proposed a new method for antibody detection but also demonstrated the potential utility of the method in real‐life diagnosis.

## 4 Future Outlook

**Figure 18:**
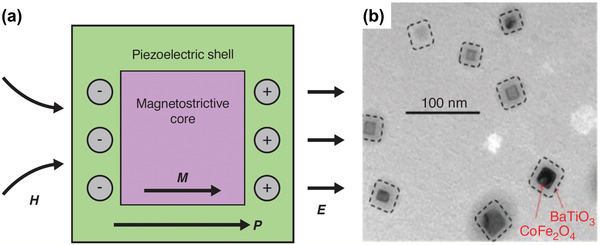
Magnetoelectric nanoparticles and their magnetic properties. a) Illustration of the basic configuration of multiferroic nanostructures. *P* and *M* are polarization and magnetization, respectively, and *E* and *H* are the respective electric and magnetic field intensities. b) TEM image of CoFe_2_O_4_–BaTiO_3_ composite. a,b) Reproduced with permission.^[^ ^127^ ^]^ Copyright 2019, Cold Spring Harbor Laboratory Press.

The research outcomes reported above have demonstrated the potential and benefits of piezoelectric and magnetostrictive materials in detecting specific viruses. **Table** **6** summarizes the various materials and methods. Near future work should further improve the accuracy and efficiency, reduce the tester size and weight, and enhance the wearability of virus sensors through the bespoke design and fabrication of multifunctional piezoelectric and magnetostrictive materials. For example, lamination of piezoelectric and magnetostrictive layers^[^ ^126^ ^]^ or dispersions of magnetoelectric (ME) nanoparticles^[^ ^127^ ^]^ (see **Figure** **18**a) might be one area of interest. Figure 18b shows a TEM of ME composite made of the magnetostrictive core CoFe_2_O_4_ and piezoelectric shell BaTiO_3_. To achieve a design optimized for sensor performance and benefits, parametric studies need to be conducted through theoretical investigation^[^ ^128^, ^129^ ^]^ including multiphysics and multiscale numerical simulation.^[^ ^130^, ^131^, ^132^ ^]^ Improving modeling accuracy could remarkably increase the efficiency of the structural optimization of computational interactions between mechanical and electromagnetic fields, thereby reducing the time and cost of manufacturing and tooling in experiments. At the same time, such enhancement would also help to determine the microscale/nanoscale mechanisms impacting both mechanical and electromagnetic behavior of the functional piezoelectric and magnetostrictive materials.

**Table 6:** Comparison of virus sensor technologies

Material	Method	Virus type	Detection limit	Detection range	Detection time	Advantage	Disadvantage/future work	Ref.
Quartz	PCR	HPV	55 ± 7.4 Hz		39 min		Unable to set constant temperature below 10 °C	^[^ ^66^ ^]^
	LAMP‐QCM	HPV‐58	34 ± 3.6 Hz		30 min	Lipid detection; fast preparation; real‐time measurement	Lower diagnostic specificity at 90.5%	^[^ ^67^ ^]^
	QCM‐D	DENV NS‐1	0.1 µg mL^−1^	0.01–10 µg mL^−1^		More sensitive, fast and less expensive diagnostic assays		^[^ ^78^ ^]^
	QCM	HIV‐1	2 ng mL^−1^			Easy preparation; high stability and sensitivity		^[^ ^88^ ^]^
	QCM	HIV‐1	1 ng mL^−1^			Low concentration limit		^[^ ^90^ ^]^
	QCM‐D	HBV	≈4.0 × 10^12^ molecules cm^−2^			Without the need of amplification steps or labeling methods	Fabrication of biosensors able to detect in a single‐step process	^[^ ^93^ ^]^
LiTaO_3_	SAW	Ebola	1.9 × 10^4^ PFU mL^−1^	1.6 × 10^4^ to 6.5 × 10^6^ PFU mL^−1^		Adaptable, label‐free and rapid detection	Portability and optimization for field use	^[^ ^81^ ^]^
	SAW	HIV‐1; HIV‐2	12 50% tissue culture infective doses (TCID_50_s) for HIV‐1; 87 TCID_50_s for HIV‐2		5 min	Rapid and accurate detection	Testing the clinical sensitivity and specificity using larger cohorts of infected patient sera in blinded case‐control studies	^[^ ^89^ ^]^
SiO_2_/LiNbO_3_	SAW	Influenza A H1N1	1 ng mL^−1^			Without compensation design at room temperature		^[^ ^83^ ^]^
PZT	Piezo‐ glycopolymer receptor	Influenza A	10^5^ virions mL^−1^		15 min	Acceptable detection time; reproducibility for home appliance use		^[^ ^84^ ^]^
	Piezoreaction chamber	Anti‐HBsAg; anti‐AFP	0.1 ng mL^−1^	0.1–10 000 ng mL^−1^	Less than 2 h	Simultaneously detect eight different analytes	Price reduction due to further miniaturization and optimization	^[^ ^92^ ^]^
PMN‐PT	Piezoprobe DNA	HBVDM	60 copies mL^−1^		30 min			^[^ ^94^ ^]^
	Piezoprobe DNA	HBVDM; KRAS	As low as 10^−16^ mol m^−3^			Highly specific in situ, amplification‐free and label‐free mutation detection		^[^ ^95^ ^]^
Metglas	Magnetostriction	CSFV	0.6 µg mL^−1^			Resonance frequency shift linearly proportional to CSFV concentration; simple circuit design; fast signal processing; visual liquid crystal display		^[^ ^124^ ^]^
	Magnetostriction	CSFV E2	2.466 ng mL^−1^		Several min	US$ 0.001 per sensor; minimum skill; smaller size		^[^ ^125^ ^]^

Here, some key guidelines are summarized for improving the performance of the piezoelectric and magnetostrictive biosensors:

1. It is desired to develop piezoelectric and magnetostrictive sensors that can detect the viruses from changes of the output voltage instead of the resonance frequency change, as mentioned in the Introduction. For the piezoelectric sensors, both driving and sensing electrodes are needed.^[^ ^47^ ^]^ The benefit of this detection method is that it does not require fast Fourier transform (FFT) or discrete Fourier transform (DFT) analysis and will shorten the detection time. In order to increase the sensitivity (e.g., output voltage change with respect to the mass change), it is necessary to investigate and identify the optimum materials and structures and vibration mode by numerical simulation.
2. As mentioned earlier, the sensitivity varies depending on the distribution^[^ ^109^ ^]^ and location^[^ ^105^ ^]^ of the virus mass. Therefore, it is recommended to simulate the effects of the distribution and location of the mass on the sensitivity of the biosensor subjected to vibration. At this time, it is important to understand the locations of the stress concentration and strain concentration of the biosensors. This knowledge will provide an effective route to the design and optimization of the biosensor.
3. By designing a composite biosensor using piezoelectric and magnetostrictive materials, it will be possible to apply it coillessly or wirelessly depending on the application or situation. Combining with soft materials may solve the brittleness of piezoelectric element and the eddy current of magnetostrictive materials (see Table 1). Furthermore, taking advantage of the characteristics of composite layered materials, it will be possible to develop biosensors that detect multiple viruses simultaneously. Material/structure optimization can be achieved by numerical simulations accounting for material and geometric nonlinearities, heterogeneous microstructure and complex shapes.

**Figure 19:**
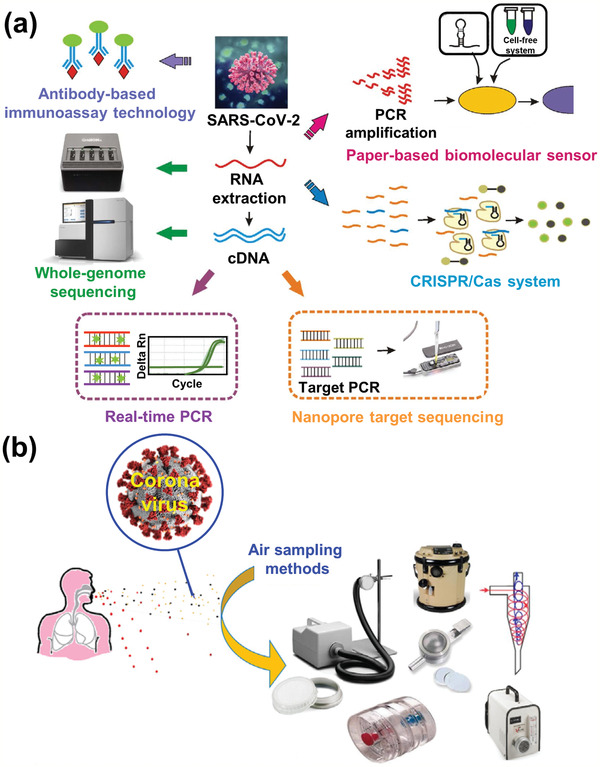
a) Developed methods for detection of novel coronavirus SARS‐CoV‐2. b) Sampling and detection of coronaviruses in air. a) Reproduced under the terms of the CC‐BY Creative Commons Attribution 4.0 International license (https://creativecommons.org/licenses/by/4.0).^[^ ^133^ ^]^ Copyright 2020, The Authors, published by John Wiley & Sons Australia, Ltd and Shanghai Fuji Technology Consulting Co., Ltd. b) Reproduced with permission.^[^ ^135^ ^]^ Copyright 2020, Elsevier.

**Figure 20:**
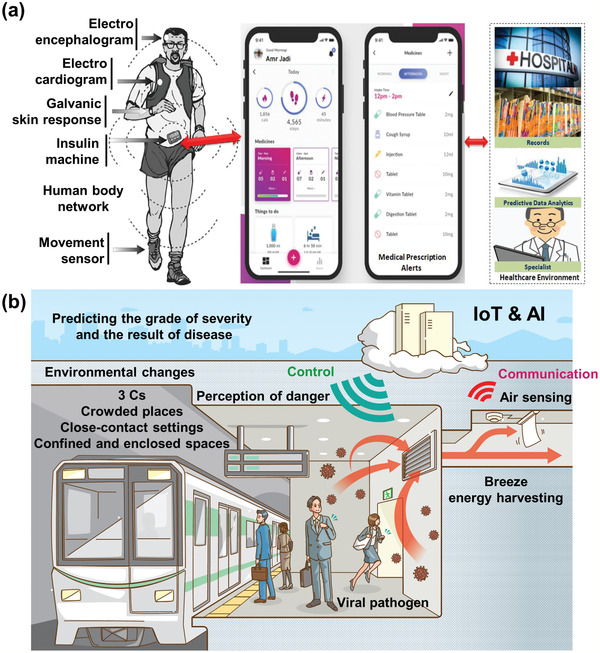
a) Proposed mobile health system. b) Proposed future society. a) Reproduced with permission.^[^ ^136^ ^]^ Copyright 2020, The Science and Information Organization.

In recent years, some new methods have been developed that can detect SARS‐CoV‐2, as shown in **Figure** **19**a.^[^ ^133^ ^]^ A new assay device for identifying both SARS‐CoV‐2 and other winter viruses including influenza A has also been developed.^[^ ^134^ ^]^ It was reported that it could successfully analyze DNA and detect SARS‐CoV‐2 within 90 min in nonclinical settings without the need for supervision by a trained healthcare professional. In addition, the detection of SARS‐CoV‐2 in air has been investigated (Figure 19b).^[^ ^135^ ^]^ It is estimated that IoT and artificial intelligence (AI) will have huge economic impacts by 2030 and will be increasingly in demand in the coming “postcorona society.” For example, IoT‐connected biosensors with AI may become ubiquitous.^[^ ^136^, ^137^ ^]^ Along similar lines, **Figure** **20**a shows a proposed mobile health system. This system can be used for patient health monitoring with a constant recording and feedback approach, but it can also be extended to track and trace infections for public health protection.

**Figure 21:**
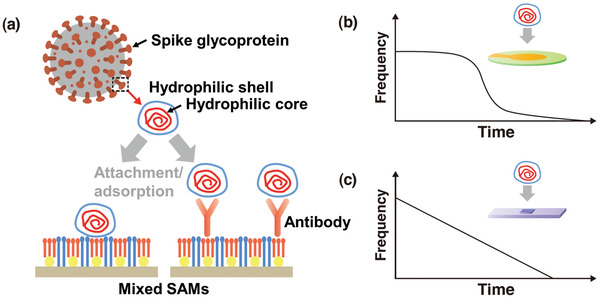
a) Schematic of biointerfacial interactions spike glycoprotein with engineered surface (mixed self‐assembled monolayers (SAMs)) alone or coupled with an antibody (antispike glycoprotein). b) Frequency to time during SARS‐CoV‐2 detection using quartz crystal microbalance (QCM). c) Frequency to time during SARS‐CoV‐2 detection using magnetostrictive microcantilever (MSMC). In (b) and (c), engineered surfaces are omitted and not drawn. a,b) Reproduced with permission.^[^ ^139^ ^]^ Copyright 2020, Taylor & Francis.

Currently, the development of efficient and reliable piezoelectric and magnetostrictive based biosensors that detect SARS‐CoV‐2 is desired. In fact, the detection of SARS‐CoV using the piezoelectric immunosensor has been reported,^[^ ^138^ ^]^ and implementation of the recently proposed surface chemistry on the quartz crystal surface of the QCM will allow the early detection of SARS‐CoV‐2 (see **Figure** **21**a,b).^[^ ^139^ ^]^ Exploration to implement these engineered surfaces on the MSMC surface is needed (Figure 21c). Both piezoelectric and magnetostrictive materials are also promising candidates for energy‐harvesting from ambient environmental sources to self‐power biosensors for virus detection and communication. Such an advance would open a novel avenue for autonomous, digitalized virus detection. It is desirable to integrate wearable biosensors with energy‐harvesters as self‐powering sources. Such multifunctional self‐powered sensing systems can potentially manage health by automatically accumulating and transmitting data, contributing greatly to the realization of a society in which people can live without fear of infection by various viruses. Figure 20b shows an example of such a future society. The connected IoT biosensor detects viral pathogens in the air. From the big data provided by numerous IoT biosensors, the spread of infection can be understood in real time, and its short‐term and long‐term impacts both locally and globally can be modeled in real time. Wearable actuators may be able to direct people in hazardous areas to escape. The power used may be harvested from the breeze flowing^[^ ^23^ ^]^ through the air conditioning duct for 24 h in case of houses and underground malls. AI can potentially predict a disease's degree of spreading and severity. It is necessary for material researchers to cooperate with researchers from electrical/electronics and computer science fields to advance interdisciplinary investigation and better protect communities from various infectious diseases.

## 5 Concluding Remarks

Herein, we have systematically summarized and reported progress in piezoelectric and magnetostrictive materials applied to biosensors. Based on the published literature we collected, these materials show great potential to be used in the detection of various infectious viruses. In particular, sampling and detection of coronavirus in the air were discussed and are currently under intensive investigation. The intention of the article is to guide and assist material researchers to reexamine the design and performance of existing piezoelectric and magnetostrictive biosensors. It is hoped that the study will inform and assist these researchers to develop more effective and reliable sensors for virus detection with higher sensitivity and accuracy (ng per mL), smaller size and weight, and affordability, for home application or wearability in the near future (smart clothing). Such virus detection sensors will become reality with the further development of materials science and technological progress in AI, machine learning and data analytics.

## Conflict of Interest

The authors declare no conflict of interest.
